# Transmission from seed to seedling and elimination of alfalfa viruses

**DOI:** 10.3389/fpls.2024.1330219

**Published:** 2024-06-06

**Authors:** Jin Li, Qiaoxia Shang, Yingning Luo, Shuhua Wei, Chaoyang Zhao, Liping Ban

**Affiliations:** ^1^ College of Grassland Science and Technology, China Agricultural University, Beijing, China; ^2^ Sanya Institute, China Agricultural University, Sanya, China; ^3^ College of Bioscience and Resource Environment, Beijing University of Agriculture, Beijing, China; ^4^ Key Laboratory of Urban Agriculture in North China, Ministry of Agriculture and Rural Affairs, Beijing University of Agriculture, Beijing, China; ^5^ Institute of Plant Protection, Ningxia Academy of Agriculture and Forestry Sciences, Yinchuan, China; ^6^ Center for Medical, Agricultural and Veterinary Entomology, United States Department of Agriculture- Agricultural Research Service (USDA-ARS), Gainesville, FL, United States

**Keywords:** alfalfa viruses, transmission from seed to seedling, alfalfa mosaic virus (AMV), Medicago sativa alphapartitivirus 2 (MsAPV2), viruses elimination

## Abstract

**Introduction:**

Viral diseases have become a vital factor limiting the development of the alfalfa (*Medicago sativa*) industry. Six viruses infecting alfalfa with a high incidence rate are Alfalfa mosaic virus (AMV), Medicago sativa alphapartitivirus 1 (MsAPV1), Medicago sativa alphapartitivirus 2 (MsAPV2), Medicago sativa deltapartitivirus 1 (MsDPV1), Medicago sativa amalgavirus 1 (MsAV1), and Cnidium vein yellowing virus 1 (CnVYV1). The purpose of this study was to develop preventive measures against these viruses by investigating their transmission through alfalfa seeds.

**Methods:**

In this study, we investigated the transmission rate of alfalfa viruses from seed to seedling by PCR, determined the location of viruses in seed by dissecting seed embryos and seed coat, tracked the changes of viruses in seedlings, and finally discover effective elimination measures for alfalfa viruses from 16 measures.

**Results and discussion:**

Our results demonstrated that all these six viruses could be transmitted from alfalfa seeds to seedlings with the transmission rate ranging from 44.44% to 88.89%. For AMV, MsAPV2, and MsAV1, the viral load was significantly higher in the seed coats than in the seed embryos; however, it did not show significant differences between these two parts of the seeds for MsAPV1, MsDPV1, and CnVYV1. Dynamic accumulation analysis of AMV and MsAPV2 indicated that the viral load in plants increased continuously in the early growth stage, making it important to inactivate these viruses prior to their seed-to-seedling transmission. Sixteen treatments including physical, chemical, and combinations of physical and chemical measures were compared in terms of their elimination efficiency on AMV and MsAPV2 and impacts on seed germination. The results showed that soaking alfalfa seeds in sterile distilled water for 2h + 2% NaClO for 1h or 2% NaClO for 1h were more promisingly applicable because it could significantly reduce AMV and MsAPV2 particles in both seeds and seedlings. Our data revealed a route of virus transmission in alfalfa and shed light on the discovery of a highly efficient method for the management of alfalfa viral diseases.

## 1 Introduction

Viral diseases are one of the most significant biological threats to alfalfa (*Medicago sativa*). Currently, more than 50 viral species have been reported that can infect alfalfa, causing severe symptoms such as enation, streak, yellowing, and mosaic, and inhibiting the normal growth and development of the alfalfa (Han et al., 2019; Jiang et al., 2019; Li et al., 2021; Guo et al., 2022; Li et al., 2022). Viral diseases have become a vital factor hindering the development of the alfalfa industry. Seeds are an important source for dissemination of plant diseases, and provide an route for virus infection in plants at their early stage (Ban et al., 2021). Also, seeds may serve as the initially infected source for plant viruses to be further transmitted to other plants through insect vectors (Li et al., 2021; Liu et al., 2021; McNeill et al., 2021), resulting in the rapid epidemic of viral diseases in the field.

At present, twenty-one species of alfalfa viruses have been detected in China (Guo et al., 2022; Li et al., 2022). In our previous study, we found six viruses with a high incidence rate and virus loads in alfalfa were alfalfa mosaic virus (AMV), Medicago sativa alphapartitivirus 1 (MsAPV1), Medicago sativa alphapartitivirus 2 (MsAPV2), Medicago sativa deltapartitivirus 1 (MsDPV1), Medicago sativa amalgavirus 1 (MsAV1), and cnidium vein yellowing virus 1 (CnVYV1) (Li et al., 2022). As a worldwide spread vital alfalfa virus that has drawn extensive attention, AMV may cause alfalfa biomass loss by up to 30% (Han et al., 2019). AMV particles are attached to the seed coats in a stable form, highly resistant to potential damages caused by storage temperature and location changes (Frosheiser, 1964, Frosheiser, 1974). By contrast, the other five viruses, MsAPV1, MsAPV2, MsDPV1, MsAV1, and CnVYV1, were recently detected in the main alfalfa cultivation region in China (Li et al., 2022), but whether and how they were transmitted by seeds remained to be investigated. Moreover, virus accumulation in plants and the dynamic process vary among different viral species (Ryabinina et al., 2020; Xu et al., 2023). Exploring the dynamics and understanding the virus replication process in the early stage of plant development may provide insights into early virus diagnosis and viral disease management.

Effective management of viral diseases in plants relies on the prevention of virus spread given that little can be done to restore the health of plants once they are infected by viruses. For seed-borne plant viruses, prevention of their spreading includes the removal of infection sources by virus elimination in seeds. For example, seeds may be treated with dry heat or microwave, or soaked in a solution of trisodium phosphate (Na_3_PO_4_), hydrochloric acid (HCl), or sodium hypochlorite (NaClO) (Herrera-Vásquez et al., 2009; Ren et al., 2017; Davino et al., 2020; Samarah et al., 2021). Viruses attached to seed surfaces could be eradicated or inactivated markedly with these protocols, which are economical and effective ways to reduce viral infections. Studies have shown that Melon necrotic spot virus (MNSV) could be eradicated from melon seeds after 144h of heat treatment at 70°C (Herrera-Vásquez et al., 2009), and that heat treatment was also effective to eradicate simultaneously three main viruses, Cucumber mosaic virus (CMV), Watermelon mosaic virus (WMV), and Zucchini yellow mosaic virus (ZYMV), that co-parasite in zucchini seeds (Ren et al., 2017). Treating alfalfa seeds using 2000 mg/L of calcium hypochlorite or aqueous ozone has been shown to be effective, with an inactivation efficiency ranging from 66.03% to 82.78%, to decontaminate human viruses such as Human norovirus, Murine norovirus, and Tulane virus, all of which can cause human gastroenteritis (Qing and Kalmia, 2014; Wang et al., 2015).

In spite of numerous studies on treating seeds for virus elimination, little has been known regarding elimination against plant viruses on alfalfa seeds. Therefore, in this study, we set forth to investigate the transmission of six important alfalfa viruses via seeds and discover effective elimination measures for the control of these viral diseases on alfalfa seeds.

## 2 Materials and methods

### 2.1 Plant materials

The twelve alfalfa cultivars widely cultivated throughout China and analyzed in this study were “Caribou”, “Sanditi”, “Zhongmu No.1”, “Zhongmu No.2”, “Gannong No.3”, “Gannong No.5”, “Zhaodong”, “Juneng 601”, “WL343HQ”, “310SC”, “42IQ”, and “4030”. The seeds of these varieties are further used to extract seed total RNA, as well as to detect the six alfalfa viruses in seeds. Nine replicates per cultivar, each replicate consisting of ten randomly selected seeds, were used for virus load detection.

### 2.2 Seed dissection

Prior to separating seed coats from embryos, the seeds were soaked in sterile, deionized water for four hours. To prevent cross-contamination of viruses between different tissues, the tweezers used to handle the seeds were sterilized by 75% ethanol spraying and burning. The seed coats and embryos after dissection are further used to extract total RNA and to detect the six alfalfa viruses loads. Each replicate of sample consisted of twenty seed coats or embryos, and five replicates were prepared for each sample.

### 2.3 Seed germination

The alfalfa seeds cv. “Zhongmu No.1” was soaked in sterile deionized water for four hours, then transferred on moist filter paper in 150mm Petri dishes, which were then placed in a growth chamber (Ningbo Jiangnan Instrument Factory, Zhejiang, China) at a temperature of 26°C and a humidity of 60%. To evaluate virus transmission via seeds, seedlings were collected for virus quantification on day seven post-germination. This test comprised a total of 90 seedlings, with ten seedlings per replicate for a total of nine replicates.

To determine the germination energy and germination rate which were both used to characterizing seed germination, the progress of the germinated seeds during the germination process was observed and recorded daily. Fifty seeds were placed in a Petri dish for germination, which was repeated three times. Germination energy used to determine the germination speed was calculated as the number of seeds that germinated within the first four days divided by the total number of seeds. The germination rate was calculated as the number of seeds that germinated within the first seven days divided by the total number of seeds (Xiang et al., 2022).

### 2.4 Dynamic of viral load accumulation

To investigate the dynamic changes in the loads of AMV and MsAPV2, seeds of alfalfa cv. “Zhongmu No.1” was planted individually in a 10 cm x 10 cm pot filled with sterile nutrient substrates. The pots were then placed in a growth chamber with a photoperiod of 14 hours of light and an air temperature of 26°C. Leaves from germinated seedlings were harvested every three days for five weeks, starting when the second leaves appeared. Each replicate consisted of leaves collected from five plants with five replicates prepared. The collected leaves were stored at -80°C for RNA extraction.

### 2.5 Comparison of viruses elimination protocols in seeds

The seeds of alfalfa cv. “Zhongmu No.1” were treated using 16 different treatments (**Table 1**) to compare their efficacy of disinfecting AMV and MsAPV2. These treatments included nine physical-based treatment, four chemical-based treatment, and three combined physical and chemical treatments (**Table 1**). Untreated seeds were used as control. For each treatment experiment, a seed batch of 300 seeds was used.

**Table 1:** Sixteen seed elimination treatments (T1-T16).

No.	Treatment
Physical Only	
T1	Sterile distilled water for 2h
T2	60°C water for 1h
T3	Microwave at 600W for 1min
T4	Dry heat at 70°C for 24 h
T5	Dry heat at 70°C for 48 h
T6	Dry heat at 70°C for 72 h
T7	Dry heat at 80°C for 24 h
T8	Dry heat at 80°C for 48 h
T9	Dry heat at 80°C for 72 h
Chemical Only	
T10	10% Na_3_PO_4_ for 4h
T11	75% Ethanol solution for 1h
T12	2% HCl for 1h
T13	2% NaClO for 1h
Physical and Chemical	
T14	Sterile distilled water for 2h +10% Na_3_PO_4_ for 1h
T15	Sterile distilled water for 2h + 2% HCl for 1h
T16	Sterile distilled water for 2h + 2% NaClO for 1h

#### 2.5.1 Physical-based treatment on alfalfa seeds

Seeds submerged in sterile distilled water for 2h (T1), seeds submerged in 60°C sterile distilled water for 2h (T2), and microwave at 600W for 1min (T3) were used separately on alfalfa seeds. Six different thermal-based treatments were applied on alfalfa seeds, according to the protocols: seeds heated at 70°C for 24 h (T4), 48h (T5), 72h (T6); seeds heated 80°C for 24 h (T7), 48h (T8), 72h (T9) (Córdoba-Sellés et al., 2007; Herrera-Vásquez et al., 2009; Davino et al., 2020; Samarah et al., 2021).

#### 2.5.2 Chemical-based treatment on alfalfa seeds

Four different chemical-based treatments were tested to evaluate the possibility of eradicating viruses from seeds, according to the following protocols: seeds submerged in 10% trisodium phosphate solution (Na_3_PO_4_) for 4h (T10), seeds submerged in 75% ethanol solution for 1h (T11), seeds submerged in 2% hydrochloric acid (HCl) for 1h (T12), seeds submerged in 2% sodium hypochlorite (NaClO) for 1h (T13) (Herrera-Vásquez et al., 2009; Davino et al., 2020; Samarah et al., 2021). At the end of each treatment the seeds were washed three times for 5 min with sterile distilled water and dried on sterile absorbent paper.

#### 2.5.3 Combined physical and chemical treatments

Three different combined physical and chemical treatments were applied on seeds, according to the following protocols: seeds submerged in sterile distilled water for 2h + 10% Na_3_PO_4_ for 1h (T14), seeds submerged in sterile distilled water for 2h + 2% HCl for 1h (T15), seeds submerged in sterile distilled water for 2h + 2% NaClO for 1h (T16).

When dealing with treatments that require soaking, for each replicate of the treatment, seeds were immersed in 45 ml of the treatment solution in a 50-ml beaker and placed on a horizontal shaker (Huxi, Shanghai, China) for slow agitation at a speed of 150 rpm/min for the specified treatment durations. After immersion, seeds were rinsed and air-dried to their original moisture content on filter papers at ambient room conditions (25 ± 3°C) for 3 days.

The treated and untreated seeds were pre-germinated in Petri dishes on moist tissue paper at 26°C after the different treatments to understand if each treatment affects seed germination, in terms of percentage and time. Treated seeds and the seedlings germinated from treated seeds were then subject to virus quantification using qRT-PCR. Seed germination experiments were also performed as described in *2.3.* We conducted three times of elimination for each protocol, with each time including five replicates and each replicate consisting of 10 seeds or seedlings. Seeds or seedlings were ground individually in liquid nitrogen in 2 ml microcentrifuge tubes with a 5mm diameter and two 3mm diameter zirconia beads using a grinding machine (FOCUCY, Hunan, China).

### 2.6 RNA extraction and virus detection

Total RNA was extracted from the whole seeds, seed coats, or embryos following Chang’s protocol (Chang et al., 1993) using TRIzol Reagent (Invitrogen, Carlsbad, CA, USA). Then, 1 *µ*g of RNA was reverse transcribed to cDNA using the PrimeScript RT reagent Kit including a gDNA Eraser treatment (TaKaRa, Dalian, China) according to the manufacturer’s instructions.

The qRT-PCR analysis was carried out using GS AntiQ qRT-PCR SYBR Green Master Mix (Genesand, Beijing, China) on the qTOWER3 system (Analytik Jena AG, Jena, Germany). The primers for the viral genes are listed in **Supplementary Table 1**, and the absolute quantification was measured as described by Li et al. (2022). Each reaction mixture (25 *μ*L) contained 0.5 *μ*L (10 mM) of forward and reverse primers each, 12.5 *μ*L of GS AntiQ qRT-PCR SYBR Green Master Mix (Genesand, China), and 1 *μ*L cDNA (10ng RNA). The PCR conditions were set as 95°C for 5 min, followed by 40 cycles of 95°C for 10 s and 60°C for 30 s. At the end of the reaction, the melting curve of each gene was recorded, and the absolute gene expression was analyzed using the standard curve method based on the geometric mean of threshold cycle (Ct) values. Samples without a Ct value indicated negative qRT-PCR results.

### 2.7 Statistical analysis

Data were analyzed by analysis of variance (ANOVA) of SPSS Statistics v. 26.0 (IBM, Armonk, NY, USA). Fisher’s least significant difference (LSD; P ≤ 0.05) was calculated to compare means with significant F-test results.

## 3 Results

### 3.1 Transmission of viruses from seeds to seedlings

**Figure 1:**
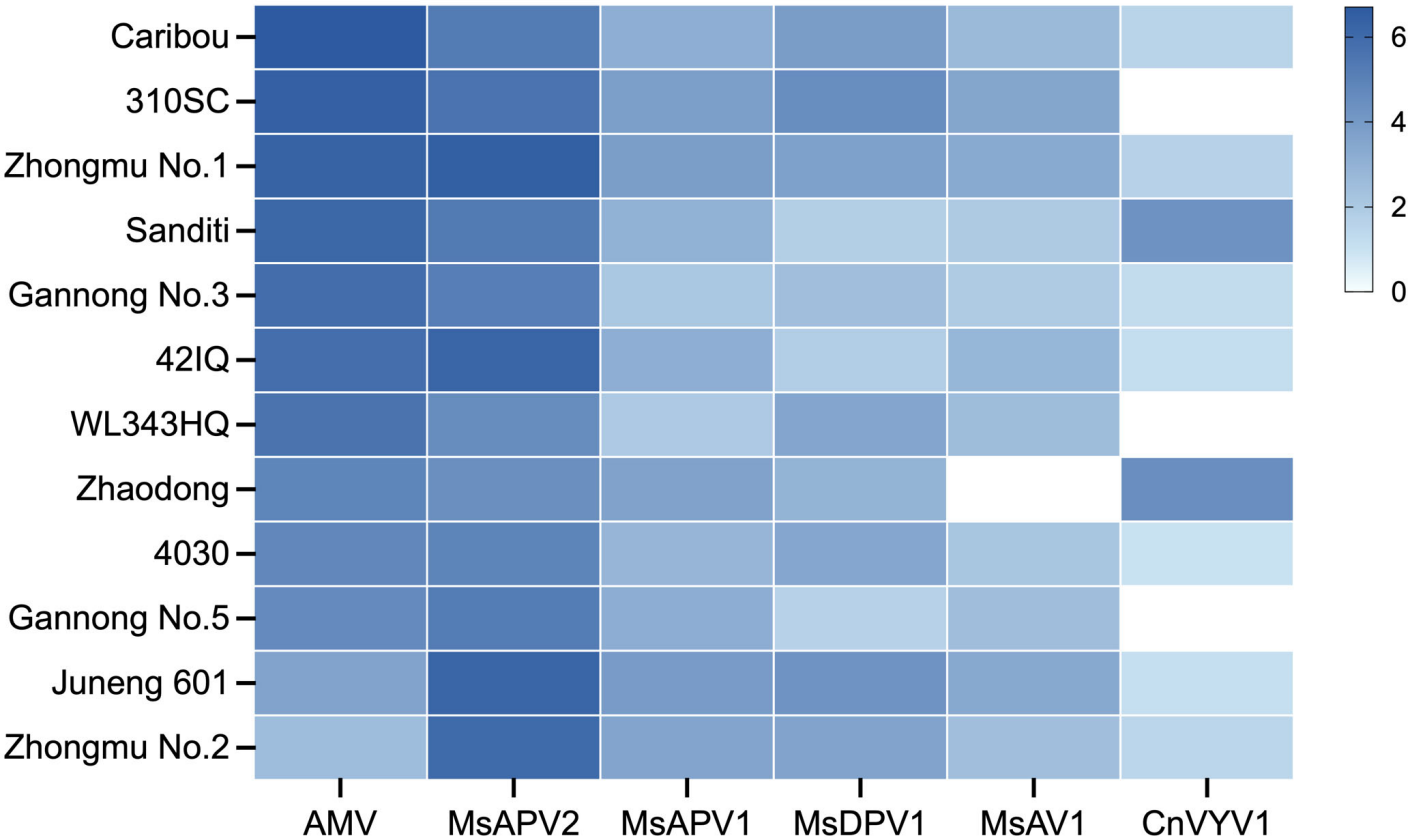
Heat map of the absolute amounts of six viruses detected in the seeds of 12 alfalfa cultivars. Virus copies were quantified by qRT-PCR. The logarithm of detected virus copies to base 10 is color coded according to the scale as shown. To ensure the representation of all samples, a value of 0.01 was assigned as the Ct value for the samples with no virus detected.

To determine whether the six predominant alfalfa viruses, i.e., AMV, MsAPV2, MsAPV1, MsDPV1, MsAV1 and CnVYV1, could be transmitted via alfalfa seeds, we conducted qRT-PCR analyses on the seeds of 12 alfalfa cultivars commonly grown in China. The results showed that all six viruses were detected in the seeds of at least eight alfalfa cultivars (**Figure 1**). All 12 cultivars were found to be positive for AMV and MsAPV2, which had higher viral accumulation than the other four viruses in the seeds of ten cultivars except the low amounts of AMV in the cultivars “Juneng 601” and “Zhongmu No. 2” (**Figure 1**). In this test, CnVYV1 was not detected in three cultivars (“310SC”, “WL343HQ”, and “Gannong No.5”), and neither was MsAV1 in “Zhaodong” (**Figure 1**).

To further determine whether these viruses could be transmitted from seeds to seedlings, we quantified these viruses in the seven-day-old alfalfa seedlings (*cv*. “Zhongmu No.1”) germinated from virus-carrying seeds by qRT-PCR and found that all six viruses were present in seedlings in spite that their copy numbers varied (**Table 2**; **Supplementary Figure 1**). Among these viruses, MsAPV2 was detected in 88.89% of seedlings, followed by AMV which was detected in 77.78% of seedlings. MsAPV1, MsDPV1, MsAV1, and CnVYV1 were found in 44.44%, 55.56%, 66.67%, and 66.67% of seedlings, respectively (**Table 2**; **Supplementary Figure 1**). We also found that the copy number of AMV in seedlings (3.75×10^2^ copies) was significantly lower (~ 5,000 times) than in seeds (2.04×10^6^ copies). On the other hand, MsAPV2 and CnVYV1 had significantly higher copy numbers in seedlings (4.73×10^7^ copies for MsAPV2, and 6.23×10^2^ copies for CnVYV1) than in seeds (2.89×10^6^ copies for MsAPV2, and 4.19×10^1^ copies for CnVYV1), while MsAPV1, MsDPV1, and MsAV1 had almost the same (no significance) number of viruses in seeds and seedlings (**Table 2**). These results indicated that alfalfa seeds may serve as a source for the six alfalfa viruses’ transmission to seedings, however, the transmission rate may vary in different viruses.

**Table 2:** Virus transmission from seeds to seedlings assessed by qRT-PCR.

Virus species	Seed infection rate (%)	Seed virus accumulation (copies)	Seedling infection rate (%)	Seedling virus accumulation (copies)	Significance
AMV	100	2.04×10^6^± 4.85×10^5^	77.78	3.75×10^2^± 1.79×10^1^	**
MsAPV2	100	2.89×10^6^± 4.23×10^5^	88.89	4.73×10^7^± 3.82×10^6^	**
MsAPV1	100	6.97×10^3^± 9.07×10^2^	44.44	1.05×10^4^± 6.12×10^2^	ns
MsDPV1	100	5.00×10^3^± 4.89×10^2^	55.56	8.18×10^3^± 1.08×10^3^	ns
MsAV1	100	2.01×10^3^± 2.68×10^2^	66.67	3.13×10^3^± 2.66×10^2^	ns
CnVYV1	100	4.19×10^1^± 1.02×10^1^	66.67	6.23×10^2^± 6.02×10^1^	*

Statistical analysis was based on nine replicates of seeds and seedlings examined. Asterisks indicate significant differences of virus accumulation between seed and seedling (* *p*< 0.05, ** *p*< 0.01, ns: not significant). Data are mean ± SE.

### 3.2 Distribution of viruses in seed tissues

**Figure 2:**
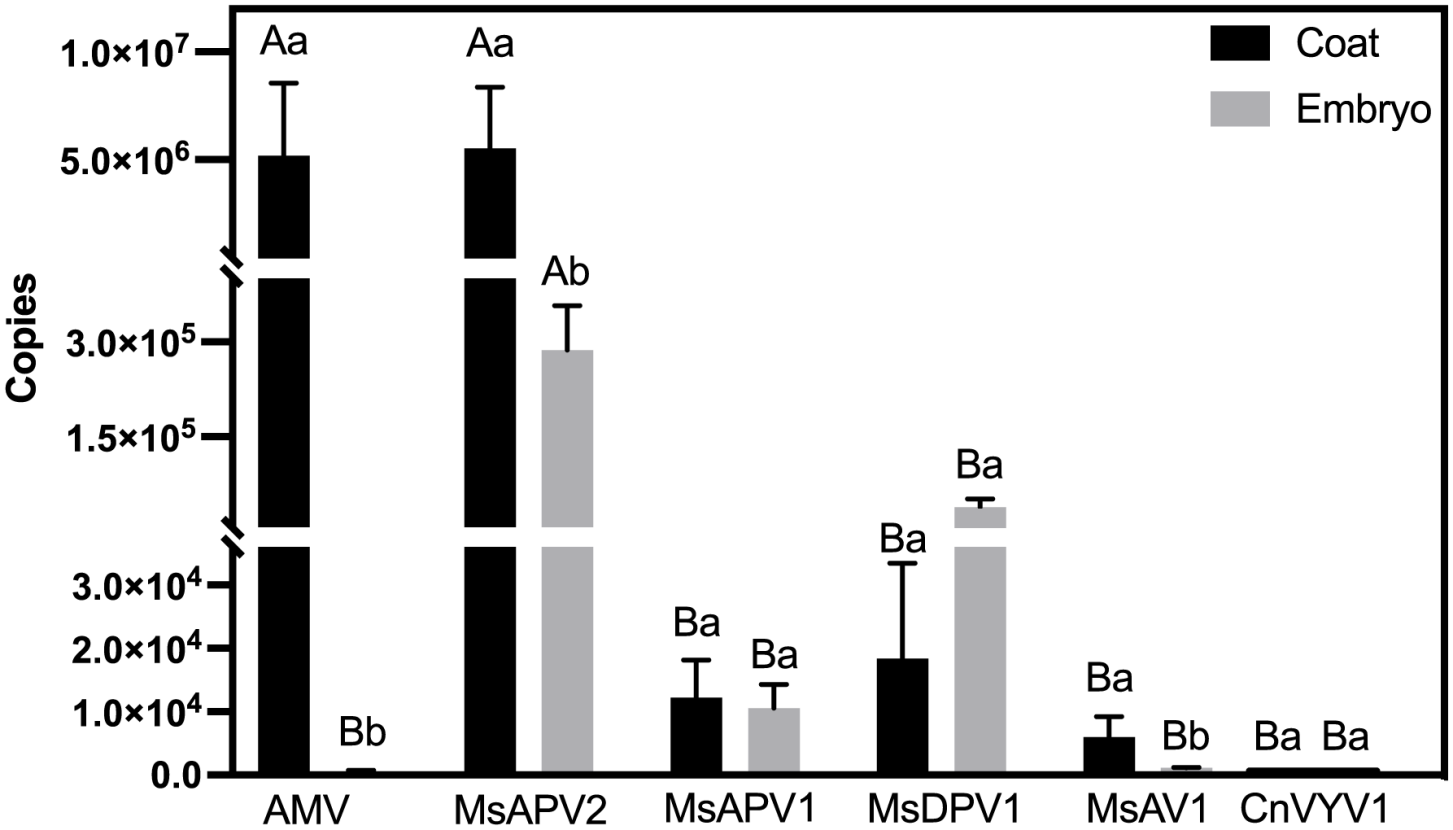
Virus titers comparison between the seed coats and embryos of alfalfa cv. “Zhongmu No.1”. Bar indicate the SE of virus concentration in four replicates, and letters “(A, B) and (a, b)” marks on the bar indicate significant differences between six treatments of coat and embryo, respectively (*p* < 0.05).

To understand how the six viruses were transmitted from seeds to seedlings, we aimed to discover which part of the seeds, the external (seed coats) or internal part (embryos), carried the viruses. All six viruses were detected in both parts of the seeds, however, their quantities differed (**Figure 2**). For AMV, MsAPV2, and MsAV1 (**Figure 2**), viral accumulation in seed coats was significantly higher than in embryos. It is worth noting that AMV in seed coats was 9209.27 times of the amount in embryos, however, MsAPV2 and MsAV1 in seed coats were only 19.24 and 5.48 times the amounts in embryos, respectively. No significant differences in virus amounts were found between coats and embryos for MsAPV1, MsDPV1, and CnVYV1, although MsAPV1 and CnVYV1 appeared more abundant in coats than in embryos, while MsDPV1 appeared more abundant in embryos than in coats (**Figure 2**).

### 3.3 Dynamics of viruses in seedlings

**Figure 3:**
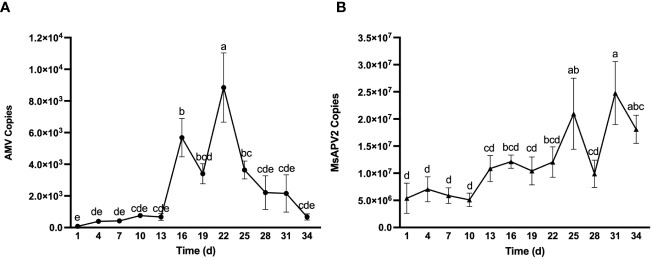
Dynamic accumulation of AMV **(A)** and MsAPV2 **(B)** in seedlings. Sampling time (X-axis) started from Day 1 when the first true leaves were unfolded. Different letters on top of error bars indicate significant (*p* < 0.5) differences of virus copy numbers.

To better understand the dynamic accumulation of viruses in seedlings, we quantified AMV and MsAPV2, the two viruses with the highest seed-to-seedling transmission rates (**Table 2**), every three days for five weeks, starting from the appearance of the first true leaf. The amount of AMV showed an upward trend at first but a downward trend later (**Figure 3A**). The initial copy numbers of AMV in seedlings were low (only 74.81 copies), but reached a peak at 8.85×10^3^ copies (with a 117-fold increase) after three weeks, and decreased to 6.69×10^2^ copies on day 34, the last day of virus monitoring. In contrast, MsAPV2 showed an overall upward trend yet with fluctuation during the whole test period (**Figure 3B**). Its initial detected copy numbers were 5.39×10^6^ copies while reaching the maximum amount of 2.48×10^7^ copies on day 31, a 4.6-fold increase compared to the initial (**Figure 3B**).

### 3.4 Elimination efficacy and effect on germination of 16 seed treatments

Based on the high incidence of virus load for AMV and MsAPV2 on alfalfa in the field (Li et al., 2022), we attempted to optimize the elimination protocol for these two viruses in this study. We tested 16 different treatments, namely T1 ~ T16 (**Table 1**), on alfalfa seeds, and then quantified AMV and MsAPV2 independently in both the treated seeds and the seedlings germinated from the treated seeds.

**Figure 4:**
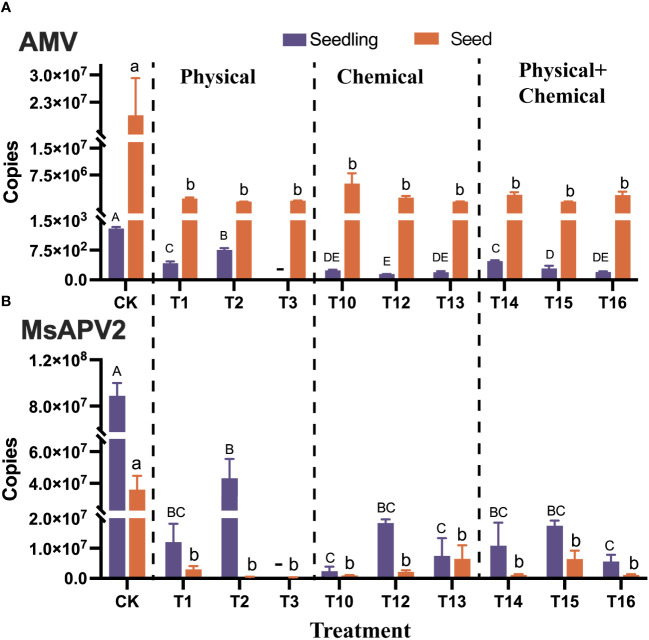
Elimination of AMV **(A)** and MsAPV2 **(B)** from alfalfa seeds using nine different treatments (T1-T3, T10, T12-T16, refer to **Table 2** for details) which effectively eliminated the viruses from alfalfa seeds and seedlings. Detected virus copies in the seedlings germinated from the treated seeds and in the treated seeds are shown on the left and right, respectively. Different uppercase or lowercase letters next to error bars indicate significant (*p* < 0.05) differences in viruses in seedling or seeds, respectively. “-” indicated the data unavailable in this treatment.

The results showed that all 16 treatments reduced AMV accumulation in seeds significantly (*p* < 0.01) from 1.89×10^7^ copies (control) to 8.41×10^4^ ~ 5.11×10^6^ copies, with a elimination efficiency of 72.93% ~ 99.55% (**Supplementary Figure 2A**, **Supplementary Table 1**). Among these, T7 (Dry heat at 80°C for 24 h), T15 (Sterile distilled water for 2h + 2% HCl for 1h), T13 (2% NaClO for 1h) and T2 (60°C water for 1h) tend to have a higher elimination efficiencies of 99.08%, 99.40%, 99.52%, and 99.55%, respectively (**Figure 4A**; **Supplementary Figure 2A**, **Supplementary Table 1**).

Virus quantification in the seedlings germinated from the treated seeds showed that eight methods significantly decreased (*p* < 0.01) AMV copies in seedlings from 1298.49 to a range of 145.79 ~ 758.06, with elimination efficiencies ranging from 41.62% to 88.77% (**Figure 4A**). Among these, the four most efficient methods were T10, T16, T13, and T12, with elimination efficiencies of 81.84%, 84.64%, 85.01%, and 88.77%, respectively (**Figure 4A**; **Supplementary Table 1**). Interestingly, seven physical methods (T4, T5, T6, T7, T8, T9, and T11) did not reduce AMV copy numbers in seedlings, instead, they increased the latter by 1.43 to 10.45 times (**Supplementary Figure 2A**, **Supplementary Table 1**).

Regarding the elimination effect on MsAPV2, all 16 protocols significantly (*p* < 0.01) decreased MsAPV2 accumulation in seeds from 3.6×10^7^ copies (control) to 2.78×10^5^ ~ 6.54×10^6^ copies, with elimination efficiencies ranging from 81.84% to 99.23% (**Figure 4B**. **Supplementary Figure 2B**, **Supplementary Table 1**). Fifteen protocols except T3 were also able to decrease MsAPV2 accumulation in the seedlings germinated from the treated seeds significantly (*p* < 0.01), from 8.88×10^7^ (control) to a range of 2.40×10^6^ ~ 4.32×10^7^ copies, with elimination efficiencies of 48.28% ~ 97.30% (**Figure 4B**; **Supplementary Figure 2B**, **Supplementary Table 1**). The six more efficient methods (elimination efficiency > 90%) were T10, T16, T5, T9, T8, and T13, which had elimination efficiencies of 97.30%, 93.72%, 92.02%, 91.85%, 91.77%, and 91.55%, respectively (**Figure 4B**; **Supplementary Table 1**).

**Figure 5:**
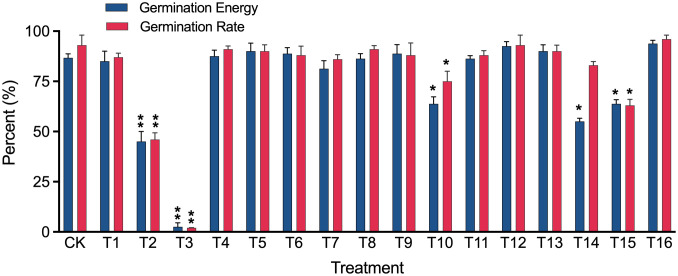
Germination energy and germination rate of alfalfa cv. “Zhongmu No.1” seeds disinfected using 16 different treatments (T1-T16, refer to **Table 2** for details). Significant (*p* < 0.05) and extremely significant (*p* < 0.01) differences from the untreated seeds (CK) are indicated by single and double asterisks, respectively, shown on top of error bars.

We also assessed the treatment effects on alfalfa seed germination rate and found that eleven out of sixteen methods had no significant impact on germination energy or rate compared to the control (CK). However, three methods (T10, T14, and T15) decreased the germination rate significantly (*p* < 0.05), and two methods (T2 and T3) decreased the germination rate extremely significantly (*p* < 0.01) (**Figure 5**).

## 4 Discussion

Seed transmission is one of the vital routes for viruses to infect their host plants, and more than 20 viral species have been found to infect alfalfa in China (Guo et al., 2022; Li et al., 2022). However, the distribution and transmission of these viruses via seeds have been rarely reported. To understand the prevalence and distribution of these viruses and select local disease-resistant/tolerant alfalfa cultivars, it is important to understand whether seeds serve as a transmission route for these viruses. This study found that all six main alfalfa viruses investigated were seed-borne viruses. We also compared 16 seed elimination protocols regarding their virus elimination efficiency and effect on seed germination for the selection of an effective protocol that can be field applied.

By performing qRT-PCR analyses, we found that the rate of AMV transmission from seeds to seedlings in alfalfa was 77.78% (**Table 2**), which was much higher than a previously reported rate (< 10%) (Frosheiser, 1964). This difference could be owing to a higher seed viral survival rate and seed transmission rate today resulting from the increase in temperature and light intensity caused by global warming (Roossinck, 2015; Bueso et al., 2017; Pagán, 2022). It is also likely owing to the highly improved sensitivity by using qRT-PCR in our work for virus detection and quantification – studies have shown that qRT-PCR is 100 ~ 1,000 times more sensitive than traditional virus quantification treatments (Torre et al., 2020; Li et al., 2022). Alternatively, there is the possibility that alfalfa cultivar may affect the seed transmission rate of the virus, such as 2.1% for alfalfa cv. Vernal and 0.8% for cv. California Common (Frosheiser, 1974). Of course, the strain of virus also affects its ability to spread (Klose et al., 1996).

Distribution analysis demonstrated that AMV copy numbers in seed coats (> 99%) were 9209.27 times of those in seed embryos (< 1%) (**Figure 2**), in alignment with a previous report which also showed that AMV was more frequently detected in seed coats than in embryos (Pesic and Hiruki, 1986). This might account for its low seed-to-seedling transmission rate because viruses on seed coats tend to be transmitted to seedlings passively through mechanical abrasion (Broadbent, 1965; Johansen et al., 1994) and become less active during the process of plant development (Johansen et al., 1994). In contrast, MsAPV2, which had a similar total quantity of virus copies in seeds (2.89 × 10^6^ copies, including those in both coats and embryos) as AMV (2.04 × 10^6^ copies) but appeared to be more enriched in embryos (2.87 × 10^5^ copies) than AMV (5.62 × 10^2^ copies), was also more abundantly detected in seedlings (4.73×10^7^ copies) than the latter (3.75×10^2^ copies). This further supports that seed embryos, rather than seed coats, may play an important role in the transmission of plant viruses from seeds to seedlings. Previous studies have demonstrated that virus-infected seed embryos could produce diseased seedlings (Uyemoto and Grogan, 1977; Wang and Maule, 1992), and seed embryos may serve as a main route of seed-to-seedling transmission because viruses that invade/infect embryos can proragate actively via replication with the development of plants from seeds to seedlings (Johansen et al., 1994).

In this study, we report for the first time that five alfalfa viruses (MsAPV2, MsAPV1, MsDPV1, MsAV1, and CnVYV1) can be transmitted from seeds to seedlings and found that their transmission rates were relatively high (44.44% ~ 88.89%). There is also a shortcoming in our study, that is, we only studied the transmission rate of the virus from seed to seedling in the market, not maternal transmission. Some of the viruses we studied have reported maternal transmission rates in the same family. The viruses of Partiviridae and Amalgavirdae are unlikely to be transmitted by vectors due to the absence of movement proteins, therefore, their spread among plants mainly relied on pollens or seeds, a means with a relatively high transmission rate (Nibert et al., 2014). For example, white clover cryptic virus-1 (WCCV-1) of Partitiviridae had a transmission rate of 48% (Guy and Gerard, 2016), and southern tomato virus (STV) and blueberry latent virus (BBLV) of Amalgavirdae had the transmission rate of 86% and 100%, respectively (Fukuhara et al., 2020; Martin et al., 2011). Unlike Partiviridae and Amalgavirdae viruses, Secoviridae viruses could be transmitted in a variety of ways, by seeds, pollens, and arthropod vectors, with varying transmission rates (Thompson et al., 2017). However, The exact maternal transmission rates of these viruses we studied is still uncertain and remains to be investigated.

We also observed that AMV and MsAPV2 demonstrated distinct dynamic accumulation patterns in alfalfa seedlings. AMV accumulation accelerated in the first three weeks of monitoring but dropped afterward until the end of observation (**Figure 3**), a pattern like the accumulation of tomato spotted wilt virus (TSWV) in infected pepper plants (Xu et al., 2023). TSWV accumulation was reported to display an increasing trend until reaching its peak at the end of the second week after inoculation, approximately 50 times higher than the amount detected on the first day, and then decrease in the following three weeks (Xu et al., 2023). This suggested that early diagnosis and elimination of plant viruses is critical for the management of these viral diseases. The decrease of viral load in plants following an increasing trend might be attributed to the activation of plant immune mechanisms as responses to mitigate viral infection. In contrast, MsAPV2, which was transmitted from seeds to seedlings at a substantial amount, demonstrated an increasing trend of viral accumulation in seedlings until the end of observation (**Figure 3**), likely as a result of continuous virus replication and accountable for the high prevalence of MsAPV2 in the field (Li et al., 2022). This resembled cucumber green mottle mosaic virus (CGMMV), whose copy numbers on the infected cucumber plants could increase continuously by ten times within five weeks (Ryabinina et al., 2020), suggesting that elimination measures must be taken in the early growth stages of alfalfa to minimize viral load on the plants and prevent MsAPV2 outbreak in the field.

To mitigate and even eradicate the infection of seed-borne viruses in alfalfa, seed treatments shall be conducted using physical and/or chemical treatments for the preparation of virus-free seeds. We compared 16 methods (T1-T16) regarding their efficacy of virus elimination and effects on seed germination and found that both T13 (2% NaClO for 1 h) and T16 (soaking in sterile water for 2h + 2% NaClO for 1h) reduced the load of AMV and MsAPV2 effectively, meanwhile improving seed germination rate, though not significantly. Similar protocols have been successfully employed to eliminate other plant viruses, such as tomato brown rugose fruit virus (TBRFV) and pepino mosaic virus (PepMV), without impairing seed germination (Córdoba-Sellés et al., 2007; Davino et al., 2020; Samarah et al., 2021). Thermal treatment has been used to eliminate viruses such as MNSV (Herrera-Vásquez et al., 2009), CMV, WMV, and ZYMV (Ren et al., 2017) by heating seeds at a minimum of 65°C. In our study, thermal methods (70°C and 80°C) did not reduce AMV copy numbers in seedlings, instead, they increased the latter. These suggested that heat treatment may not be suitable for AMV elimination. It should be noted that all 16 treatments were only able to reduce, but not eradicate AMV and MsAPV2 infection, because these viruses existed not only on the seed coats but also in the embryos (Herrera-Vásquez et al., 2009) and these treatments might only be able to inactivate virus particles attached to the seed coats or embryo surface but not to those that have already infected the embryo tissues inside. Therefore, these methods should be used together with other measures to better control these virus diseases.

Seed trade is becoming more frequent across the globe. The circulation of infected seeds will rapidly increase the spread of the virus in the world. Our study confirmed that six important alfalfa viruses can be transmitted through alfalfa seeds. Therefore, in order to prevent the spread of the virus, virus inspection and quarantine in the importing country and virus elimination in seeds in the exporting country are crucial. Further, in order to more effectively verify the elimination effect in our experiment, field experiments could be used to verify the effect, and then promoted to a larger area.

## Data availability statement

The original contributions presented in the study are included in the article/**Supplementary Material**. Further inquiries can be directed to the corresponding author.

## Author contributions

JL: Conceptualization, Formal analysis, Methodology, Writing – original draft, Writing – review & editing. QS: Methodology, Writing – review & editing. YL: Formal analysis, Methodology, Writing – original draft. SW: Formal analysis, Writing – review & editing. CZ: Formal analysis, Writing – review & editing. LB: Conceptualization, Project administration, Writing – original draft, Writing – review & editing.
