# Supplementary material for: Transmission from seed to seedling and elimination of alfalfa viruses
Source: Front Plant Sci. 2024 Jun 6;15:1330219. doi: 10.3389/fpls.2024.1330219 (PMC11187482; doi:10.3389/fpls.2024.1330219)
Supplement: Supplementary file 1 [file DataSheet_1.pdf]

Supplementary Material

1 Supplementary Figures

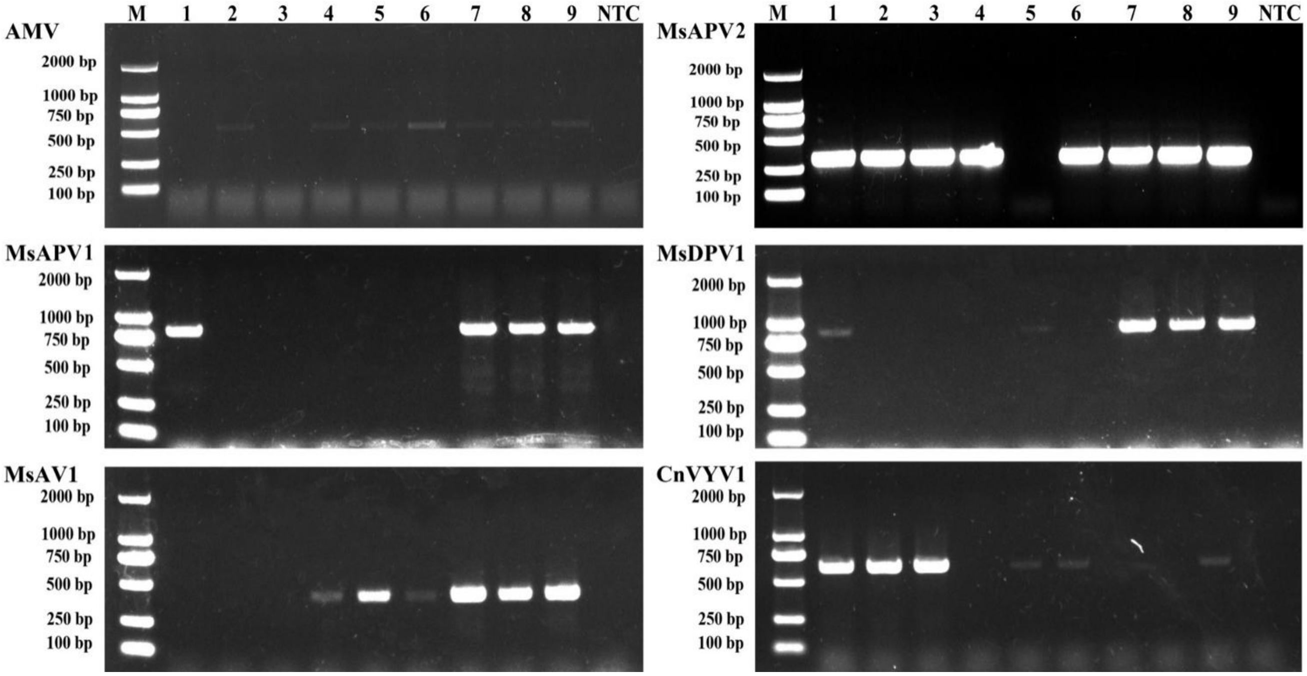

Supplementary Figure 1. PCR analysis of virus transmission from alfalfa seeds to seedlings. DNA Marker (M) and virus amplicons from nine replicates of seedlings (1-9) and no template control (NTC) are shown.

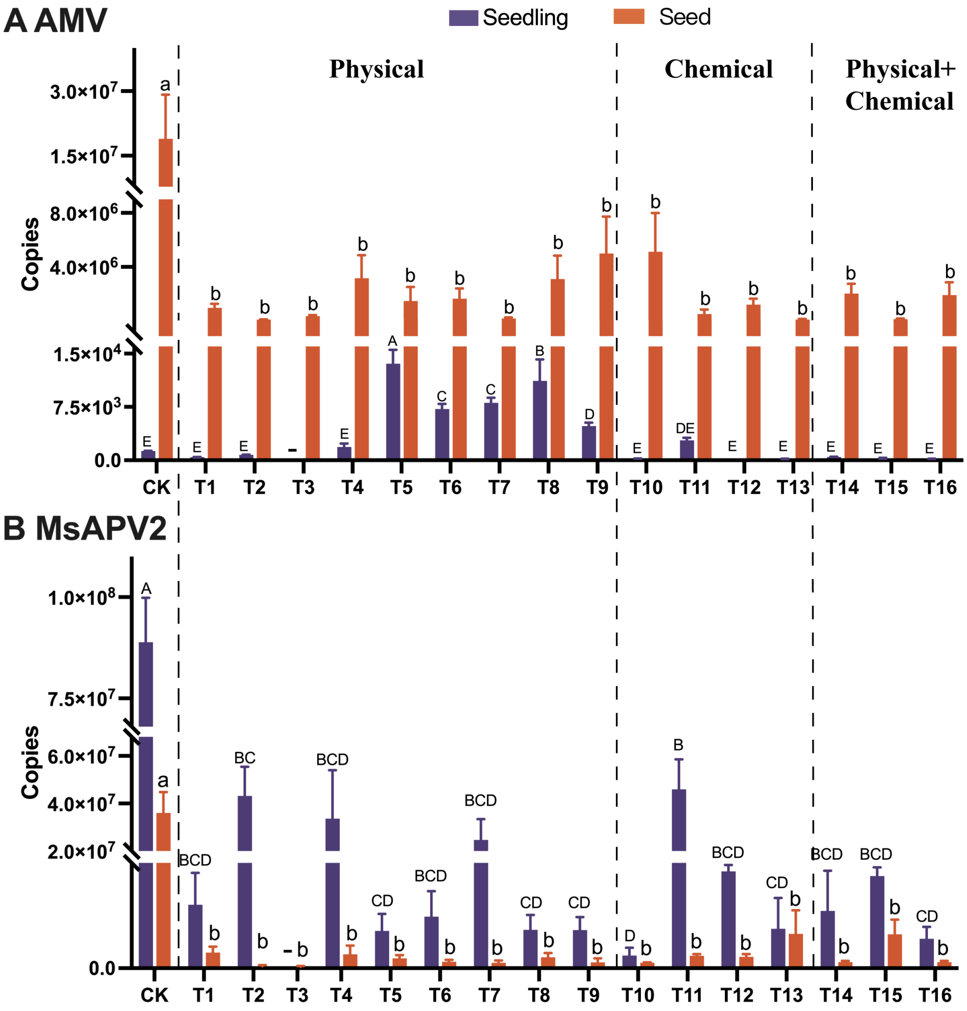

Supplementary Figure 2. Elimination of AMV (A) and MsAPV2 (B) from alfalfa seeds using 16 different treatments. Detected virus copies in the seedlings germinated from the treated seeds and in the treated seeds are shown on the left and right, respectively. Different uppercase or lowercase letters next to error bars indicate significant ($p < 0.05$) differences in viruses in seedling or seeds, respectively.

2 Supplementary Tables

Supplementary table 1. The virus elimination efficiency of each treatment

Treatment	Seed elimination efficiency of AMV	Seedling elimination efficiency of AMV	Seed elimination efficiency of MsAPV2	Seedling elimination efficiency of MsAPV2
T1	94.90%	67.69%	91.75%	86.45%
T2	99.56%	41.62%	98.89%	51.37%
T3	98.21%	100.00%	99.23%	100.00%
T4	83.33%	-43.17%	92.61%	62.16%
T5	92.28%	-945.29%	94.78%	92.02%
T6	91.38%	-453.97%	96.56%	88.96%
T7	99.08%	-520.93%	97.08%	72.24%
T8	83.70%	-759.66%	94.17%	91.77%
T9	73.60%	-270.05%	96.86%	91.85%
T10	72.96%	81.74%	97.17%	97.30%
T11	97.37%	-115.75%	93.39%	48.28%
**T12**	**93.70%**	**88.77%**	**94.03%**	**79.29%**
**T13**	**99.52%**	**85.01%**	**81.83%**	**91.55%**
T14	89.31%	63.58%	96.75%	87.73%
T15	99.40%	77.76%	82.11%	80.31%
**T16**	**89.89%**	**84.64%**	**96.78%**	**93.72%**
